# Quantitative trait loci on chromosome 1 for cataract and AMD-like retinopathy in senescence-accelerated OXYS rats

**DOI:** 10.18632/aging.100427

**Published:** 2012-01-31

**Authors:** Elena E. Korbolina, Ouyna S. Kozhevnikova, Nataliya A. Stefanova, Natalia G. Kolosova

**Affiliations:** Institute of Cytology and Genetics SB RAS, 630090, Novosibirsk, Russia

**Keywords:** age-related macular degeneration, cataract, senescence-accelerated OXYS rats, QTL analysis, congenic strains

## Abstract

Age-related macular degeneration (AMD) and cataract are common age-related diseases in humans. Previously we showed that senescence-accelerated OXYS rats develop retinopathy and cataract, which are comparable to human AMD and senile cataract. Here we focused on the identification of quantitative trait loci (QTLs), which affect early-onset cataract and retinopathy in OXYS rats, using F2 hybrids bred by a reciprocal cross (OXYS×WAG and WAG×OXYS). Chromosome 1 showed significant associations between retinopathy and loci in the regions of markers D1Rat30 and D1Rat219 (QTL1) as well as D1Rat219 and D1Rat81 (QTL2); and between early cataract development with the locus in the region of the markers D1Rat219 and D1Rat81 (QTL2). To determine the effects of these QTLs, we generated two congenic strains by transferring chromosome 1 regions from OXYS into WAG background. Both congenic strains (named WAG/OXYS-1.1 and WAG/OXYS-1.2, respectively) display early cataract and retinopathy development. Thus, we confirmed that genes located in the analyzed regions of chromosome 1 are associated with the development of these diseases in OXYS rats.

## INTRODUCTION

Age-related macular degeneration (AMD) and cataract are the most frequent eye disorders in elderly people worldwide [8, 31]. Development of cataract and AMD is influenced by a large number of genetic and environmental factors [29, 32], which remain poorly understood. Whereas early stages of these diseases cannot be studied in humans, existing animal models of complex multifactorial diseases (such as cataract and AMD) are not sufficient because of the complexity of their genetic background and/or deleterious effects of environmental factors [7, 24, 36].

We have shown previously that senescence-accelerated OXYS rats develop both retinopathy and cataracts with clinical, morphological and molecular features similar to human AMD and senile cataract [13, 14, 18, 28, 39]. This animal model is successfully used to test therapeutic action of drugs [9, 19, 20, 22, 35]. Nevertheless, the genetic basis of cataract and retinopathy development in OXYS rats has not been investigated.

A common approach to studying the genetic basis of polygenic traits, such as behavior, cataract, AMD and arterial hypertension, is to use the Quantitative Trait Locus (QTL) method, which allows for identification of the specific chromosomal loci controlling the trait of interest [3, 6, 12, 25, 26, 27, 37]. In short, the genomes of experimental animals, belonging to genetically diverse populations, are labeled with polymorphic (for instance, microsatellite) DNA markers. Then the hybrid populations are studied for associations of the marked regions (loci) of chromosomes with the phenotypic trait of interest in segregating crosses. Statistical evaluation of the significance of a marked locus's influence on the phenotypic trait is performed via calculation of a plausibility criterion (the LOD score).

We recently identified the loci that are associated with some manifestations of accelerated senescence [23]. Here we present results of 1) QTL analysis of chromosome 1 loci responsible for the development of cataract and retinopathy using the population of F2 hybrids bred by a reciprocal cross (OXYS×WAG and WAG×OXYS) and 2) generation of congenic strains by transferring two QTL regions of chromosome 1, from OXYS into the WAG background.

## RESULTS

### Cataract and retinopathy incidence in the parental population and F1, F2 hybrid rats

Manifestations of cataract and retinopathy are absent in the eyes of OXYS rats at the age of 20 days. The first AMD-like alterations in retina and signs of cataract develop in 20-22% of OXYS rats when they reach the age of 1.5 months. In recent years, the morbidity of both cataract and retinopathy has reached 100% by the age of 3-4 months. Alterations of retina are manifested as distorted reflectance of the eye ground, swelling of the retina, the emergence of distinct foci of ischemia, and the signs of atrophy of choriocapillaris and retinal pigmented epithelium (RPE). These correspond to the first stage of AMD [18, 39]. The majority of animals at this age are also characterized by lens alterations corresponding to stage 1 of cataract and exhibit zonal cortical or nuclear opacities, but 25-40% of OXYS rats already have a second stage cataract [28]. When the generation of hybrids was started, the morbidity of OXYS rats was somewhat different. The results of ophthalmoscopic examination of the parental animals are shown in Table 1. The incidence of cataracts in 3-month-old OXYS rats was independent of sex according to the data, while the incidence of retinopathy was ~ 1.3 times higher in females. This is consistent with published data on the development of AMD in humans.

**Table 1:** The incidence of cataract and retinopathy in parental OXYS rats and in rats of F1 and F2 hybrid populations at the age of 3 months

Rats	n	Cataract		Retinopathy	
		male	female	male	female
**OXYS**	61	1.31±0.096 (n = 36)	1.56±0.10 (n = 25) F(_1.59_) = 3.2 p = 0.08	0.89±0.087 (n = 36)	1.16±0.07* (n = 25) F(_1.59_) = 12.9 p = 0.03
**F1**	44	0.333±0.098 (n = 24)	1.10±0.124* (n = 20) p = 0.003	0.208±0.085 (n = 24)	0.900±0.123* (n = 20) p = 0.001
**F2**	105	0.981±0.075^#^ (n = 52) F(_1.75_) = 25.1 p <0.0001	1.340±0.147 (n = 53)	1.340±0.147^#^ (n = 52) F(_1.75_) = 24.8 p <0.0001	1.404±0.192 (n = 47)

No significant differences in the disease severity were found in the reciprocal progeny; therefore the results for WAGxOXYS and OXYSxWAG progeny were combined for the F1 and F2 populations. Data (mean ± S.E.M.) are presented as the average level of cataract or retinopathy in OXYS rats' eyes (0-3 corresponding to a stage of a disease, as explained in Methods). Legend: * – significant sex differences within one population; # – significant differences with OXYS rats.

The results of the examination of F1 and F2 hybrids are presented in Tables 1 and 2. In F1 hybrids significant sex differences were also observed, the incidence of cataracts was 3.1, and retinopathy 3.7 times higher in females than in males. The influence of gender on the incidence of ocular pathologies was confirmed by regression analysis (for retinopathy: R = 0.546, R^2^ = 0.300, adjusted R^2^ = 0.281; F (1.37) = 15.842, p <0.00031; for cataract: R = 0.587, R^2^ = 0.345, F (1.37) = 19.5, p <0.00009). There were no significant differences in the disease severity between the groups F1 (female OXYS × male WAG) and F1 (female WAG × male OXYS) (Table 2). Therefore, the data from the studies of reciprocal F1 hybrids were combined (Table 1). After that, the sex differences became even more significant: F(1.42) = 24.2, p = 0.00001 (for cataract) and F(1.42) = 22.5, p = 0.00002 (for retinopathy). In the second-generation (F2) hybrid population, the morbidity of both cataract and retinopathy significantly increased compared to the first generation (F1) only in male rats, and there were no sex differences (Table 2).

**Table 2:** The incidence of cataract and retinopathy in rats of the F1 and F2 hybrid populations at the age of 3 months

**Cataract**				
**F1**	WAG×OXYS		OXYS×WAG	
	male	female	male	female
	0.333±0.167 (n=9)	1.25±0.179♦ F(_1.19_)=13.1 p=0.002 (n=12)	0.333±0.125 (n=15)	0.875±0.125♦ F(_1.21_)=7.6 p=0.012 (n=8)
**F2**	1.05±0.135* (n=21) F(_1.28_)=9.6 p=0.004	1.04±0.14 (n=23)	0.938±0.089* (n=32) F(_1.45_)=15.0 p=0.0004	1.125±0.151 (n=24)
**Retinopathy**				
**F1**	WAG×OXYS		OXYS×WAG	
	male	female	male	female
	0.222±0.147 (n=9)	1.00±0.174♦ F(_1.19_)=10.6 p=0.004 (n=12)	0.20±0.10 (n=15)	0.750±0.164♦ F(_1.21_)=8.5 p=0.008 (n=8)
**F2**	1.29±0.240* (n=21) F(_1.28_)=7.7 p=0.010	1.04±0.261 (n=23)	1.37±0.189* (n=32) F(_1.45_)=16.7 p=0.0002	1.75±0.260 (n=24)

Data (mean ± S.E.M.) are presented as the average level of cataract or retinopathy in rats' eyes (0-3 corresponding to a stage of a disease, as explained in Methods). Legend: ♦ – significant sex differences within one hybrid population; * – significant differences with F1 male rats.

### QTL analysis

**Figure 1:**
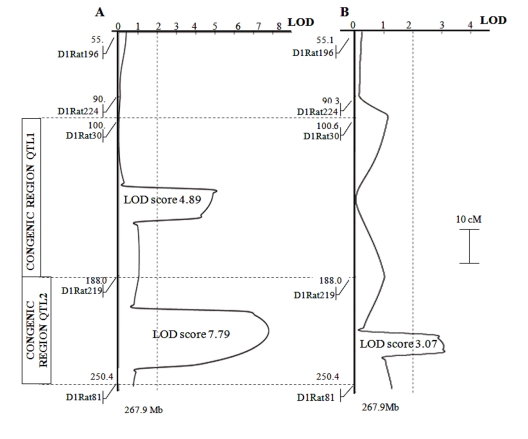
Plots of the distribution of trait linkage probability in the population of F2 rats (bred by reciprocal cross of OXYS and WAG) at age 3-4 months. The linkage of: (panel A) retinopathy; (panel B) cataract with markers on chromosome 1 was analyzed. Dotted vertical line at LOD = 2.0 is the threshold for suggestive statistical significance. The OXYS regions introgressed into WAG rats in the construction of congenic strains are shown as inserts to the left of the linkage map.

Identification of genetic loci responsible for the traits of interest was performed on hybrid F2 population at age 3-4 months. The population of 77 F2 hybrid rats showed two loci with highly significant associations of markers on chromosome 1 with the development of cataract and retinopathy. Figure 1 shows the plots of distribution of the probability of linkage between the markers analyzed and hypothetical genes controlling a trait. The locus on chromosome 1 controlling cataract development was located in the region of markers D1Rat219 and D1Rat81 (LOD score 3.07); the loci controlling retinopathy were located in the regions of markers D1Rat30 and D1Rat219 (LOD score 4.89), as well as D1Rat219 and D1Rat81 (LOD score 7.79).

### Generation of congenic rats

**Figure 2:**
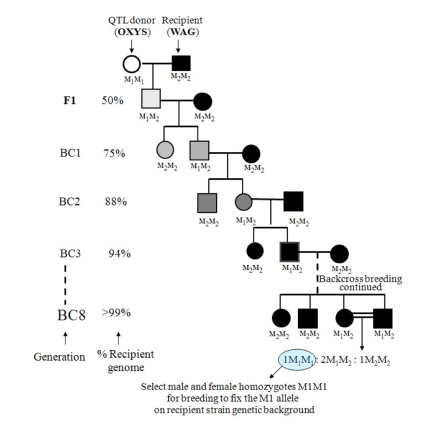
The breeding scheme for the construction of congenic strains. Selection is made at microsatellite marker locus M through a series of backcrosses such that the M_1_ allele of a donor (OXYS) replaces the M_2_ allele of a recipient (WAG). Rats are genotyped at M by PCR reaction using DNA obtained from tail tips. The genome of the donor strain is shown as an open symbol, and the genome of the recipient strain as a solid symbol. Increasing shades of gray from light to dark represent an increase in the percentage of genetic background of the recipient that occurs with each backcross (BC). The scheme is based on [26].

The construction of a congenic strain is a standard procedure of experimental mammalian genetics [4, 12, 37] originating from the work of G.D. Snell [33] on histocompatibility loci. Figure 2 outlines the procedure for moving a polymorphic marker gene from one inbred (donor) strain to another inbred (recipient) strain. In our study congenic strains were produced in accordance with the traditional approach: a QTL allele from the OXYS strain was introgressed into the WAG strain, which showed no signs of cataract and retinopathy development. The transfer of the OXYS donor region was accomplished by first producing F1 offspring by the reciprocal crosses of donor OXYS strain with recipient WAG strain (female WAG×male OXYS and female OXYS ×male WAG) and then backcrossing F1 rats with rats of a recipient WAG strain to obtain BC1 progeny. In Figure 2, the donor at marker locus M has genotype M1M1, and the recipient is M2M2. Tail tip DNA was isolated by the standard proteinase K digestion method and offspring rats were genotyped to identify animals heterozygous for a desired chromo-some 1 segment (5 markers were used: D1Rat30, D1Rat54, D1Rat219, D1Rat117, D1Rat76, and D1Rat81) and homozygous for the highest amount of recipient alleles at markers outside the chromosomal region of interest. These animals (‘carriers’) were backcrossed with new WAG rats to generate BC2 progeny for genotyping. Thus, 8 backcross generations characterized by subsequent genotyping were produced, as shown in Figure 2. This was done to ensure that nearly 99% of the donor's OXYS genetic background had been replaced by that of the recipient WAG [26]. Upon completion of these 8 backcrosses, brother-sister mating of two heterozygotes created the desired chromosomal regions homozygous for the donor's alleles and yielded offspring in the ratio M1:2M1M2:1M2M2. Two homozygous offspring M1M1 were bred to fix the M1 donor marker allele (along with a cosegregating locus of interest) in the recipient background. The rats of the two congenic strains were designated formally as WAG /OXYS-1.1 and WAG /OXYS-1.2.

The aim of the subsequent experiments was to assess the effect of these presumptive QTLs on disease manifestation in the congenic rat strains. Congenic rats serve as “proof of concept” that a QTL, when placed in another genetic background, has a meaningful effect *in vivo* on a particular phenotype [4].

### Characteristics of congenic rats

**Figure 3:**
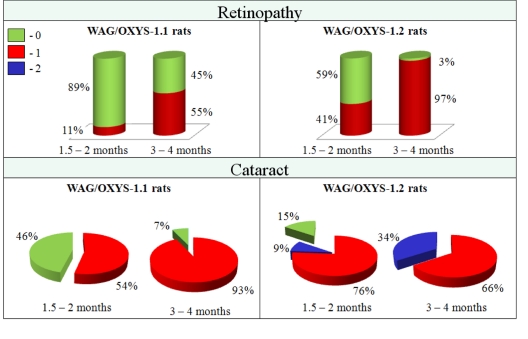
Cataract and retinopathy incidence in rats of WAG/OXYS-1.1 and WAG/OXYS-1.2 congenic strains at the ages of 1.5-2 and 3-4 months. Data are presented as a distribution of animal eyes with the stages of cataract and retinopathy (colors labeled with 0 through 2 correspond to a stage of cataract or retinopathy).

In the process of constructing congenic strains, the QTL allele from the OXYS strain was introgressed into the WAG strain, which showed no signs of cataract and retinopathy development. Two congenic regions of chromosome 1 are shown in Figure 1 as inserts to the left of the linkage map: loci marked by D1Rat30 and D1Rat219 (the region named QTL1: 100,6–188,0 Mb) and by D1Rat219 and D1Rat81 (region, named QTL2: 188,0–250,4 Mb). Figure 3 shows data on cataract and retinopathy morbidity and sickness rate for each congenic strain. The expected results were that the WAG/OXYS-1.1 congenic strain would develop retinopathy, and the WAG/OXYS-1.2 strain would develop both early cataract and retinopathy.

At the age of 1.5-2 months, in 54% of the eyes of WAG/OXYS-1.1 congenic animals, we found cortical and posterior subcapsular opacities corresponding to the first stage of cataract. For animals of the WAG/OXYS-1.2 strain the percentage was 76% of the eyes for the stage 1 cataract and 9% of the eyes for the second-stage cataract. The percentage of afflicted animals increased with age. In the group aged 3-4 months 93% of eyes were affected by the first-stage cataract in WAG/OXYS-1.1, but there were no cases of stage 2 cataract. The incidence of cataract had reached 100% in animals of WAG/OXYS -1.2 by age 3-4 months, and in 34% of the eyes the pathological changes matched the 2nd stage of the disease.

The cataract developed against the background of progressive retinopathy in congenic animals like in OXYS rats. At the age of 1.5-2 months, the signs of stage 1 retinopathy were found in 11% of the eyes of WAG/OXYS-1.1 rats. At the age of 3 months the retinopathy incidence in this strain was 55%. Interestingly, these values were approximately 2-fold lower than those in OXYS rats. In WAG/OXYS-1.2 animals at age 1.5-2 months, stage 1 retinopathy was observed in 41% of the eyes, but at 3-4 months 97% of the eyes were affected. Nonetheless, the animals of both congenic strains contained no cases of stage 2 retinopathy.

## DISCUSSION

QTL mapping approach is only the first step toward identifying the specific genes whose mutations or differential expression modulates ocular pathologies. As shown in Figure 1, the available information gives only a rough approximation of the position of the gene, or genes, in the broad chromosomal regions identified by a chromosomal or whole-genome scan. A limitation of our study is that only five markers were used to map chromosome 1 in the F2 hybrid population. Accordingly, we were able to statistically link the inheritance of disease-related phenotypes to chromosome loci of approximately 80 Mb each. The small number of appropriate markers is due to the fact that WAG and OXYS inbred strains are barely polymorphic at the majority of known microsatellite marker sites in their genomes. Thus, it is conceivable that there are other, possibly stronger genetic determinants for retinopathy that are not detectable in a cross between WAG and OXYS progenitors because these strains carry the same alleles. There are reports in literature of abnormalities in the WAG/Rij rat strain that were first thought to be an inherited retinal degeneration but later dismissed as a light-induced retinal change [17]. In this regard, the WAG strain proved useful in identifying different phenotypic variants in F2 populations because we found no signs of cataract and retinopathy in paternal rats of the WAG strain.

The number of animals examined and the fact that the sampling included both male and female rats also constrained the reliability of QTL detection in our study. As commonly reported in human, rat and murine linkage studies, loci can differentially affect males and females, and the effect of lineage is substantial too [11, 34]. In spite of all limitations and because of the high LOD score ratios observed, we supposed that the loci marked by microsatellites D1Rat30 and D1Rat219, D1Rat219 and D1Rat81 of the first chromosome include genes controlling cataract and retinopathy onset in rats of the F2 population with a high degree of probability. Subsequently we proved the existence of QTL1 and QTL2 loci on chromosome 1 by introgressing the OXYS alleles into the WAG genetic background. Both congenic strains (WAG/OXYS-1.1 and WAG/OXYS-1.2, respectively) displayed early development of cataract and retinopathy as the rats of the donor OXYS strain. In locus 2, we saw the expected result, and the morbidity of WAG/OXYS-1.2 congenic rats at the age of 1.5-2 and 3-4 months was similar to the morbidity of OXYS rats. We made an assumption at this point that QTL2 would be more promising for the study of genetic determinants of cataract and retinopathy development in OXYS rats. As for QTL1, we found that this locus had an influence on both cataract and retinopathy development in WAG/OXYS-1.1 congenic animals, although it was not associated with cataract in F2 population. Thus, we could reasonably assume that in the QTL1 locus there is a single gene or gene cluster, whose structural and/or functional defects lead to pathophysiological processes that contribute to both retinopathy and cataract.

Similar cases have been reported in literature, for example, a chromosome 4 region in mice was found to modulate both lens and iris disease [37]. Recessive mutations of the tyrosinase-related protein1 (tyrp1) gene in DBA/2J mice lead to a combination of glaucoma, cataract, anterior synechia, and iris discoloration and damage, as well as brown pigment deposition on the anterior lens surface [2].

There is also the possibility that the QTL1 locus contains two genes (gene clusters), each specific to only one of the relevant traits. But the cataract-inducing effect of this locus not observed in the regions of markers D1Rat30 and D1Rat219, possibly, due to peculiarities of the QTL analysis. Further investigation could determine whether QTL1 indeed contributes to the pleiotropic effects or if it is merely masking a significant part of the double QTL effect. Interestingly, the retinopathy incidence in rats of WAG/OXYS-1.1 congenic strain was approximately half of that in OXYS rats. Therefore, at least two retinopathy-associated genes (or gene clusters) are likely to exist on chromosome 1. It is possible that in OXYS rats the development of cataract is caused by more than one locus.

Some of the above questions can be addressed as follows. The next step could be the refinement of each QTL interval using single-nucleotide polymorphism (SNP) identification [21, 40]. The use of common inbred strains as a source of genetic variation in the segregating cross will also facilitate QTL localization. A higher polymorphism rate between donor and recipient strains will be helpful in a genome scan.

The chromosomal regions delineated in our mapping study contain genes previously found to be associated with ocular diseases in animals. QTL1 contains gene Rlbp1 (retinaldehyde-binding protein 1, rgd: 1309649, sequence position 135 Mb). Mutations of this gene have been associated with severe rod-cone dystrophy, Bothnia dystrophy (nonsyndromic autosomal recessive retinitis pigmentosa) and retinitis punctata albescens [10]. QTL2 contains gene Rom1 (retinal outer segment membrane protein 1, rgd: 1306070, sequence position 211 Mb). The Trp^182^Arg substitution in Rom1 (Rgsc1156) mouse mutants causes progressive retinal degeneration [30]. This chromosomal region also contains gene locus Bbs1 (Bardet-Biedl syndrome 1, rgd: 1307581, sequence position 207 Mb). Bardet-Biedl syndrome is a multi-organ disease presenting with retinopathy leading to blindness with impaired photoreceptor protein transport and dysfunctional synaptic transmission, according to a mouse model [1]. Another interesting gene is a Best1 (bestrophin 1, rgd: 1311656, sequence position 212 Mb). Mutations in this gene are responsible for juvenile-onset vitelliform macular dystrophy (VMD2, also known as Best macular dystrophy) and for adult-onset vitelliform macular dystrophy (AVMD) [38]. The possible candidate gene is Fxn (frataxin, rgd: 1565754, sequence position 227 Mb). This nuclear gene encodes a mitochondrial protein that participates in the regulation of iron transport and respiration in mitochondria. Expansion of intronic trinucleotide repeat GAA in the Fxn gene results in Friedreich ataxia, a mitochondrial disorder affecting multiple systems of organs, including the visual pathway [5].

In conclusion, we found that F2 rat population at age 3-4 months showed significant associations between two characteristics of OXYS rats and genetic loci on chromosome 1. The first association is between cataract and the locus near the markers D1Rat219 and D1Rat81 (LOD score 3.07). The second association is between retinopathy and the loci in the vicinity of markers D1Rat30 and D1Rat219 (LOD score 4.89), as well as D1Rat219 and D1Rat81 (LOD score 7.79). Both congenic strains, WAG/OXYS-1.1 and WAG/OXYS-1.2, display early cataract and retinopathy development as rats of the donor OXYS strain. Further histological characterization of the congenic animals is necessary, as are studies of the dynamics of morbidity with age. These data will provide a frame of reference for further investigation of ocular pathology in prematurely aging OXYS rats.

## METHODS

### Experimental animals and ophthalmoscopy examination

We used OXYS rats, rats of WAG (Wistar Albino Glaxo) strain with no signs of age-related eye diseases, F1 and F2 hybrids obtained by the reciprocal crosses (female WAG×male OXYS and female OXYS ×male WAG), and rats of backcross and congenic populations. All animals were born and reared at the Center for Genetic Resources of Laboratory Animals at the Institute of Cytology and Genetics (ICG), Siberian Branch of the Russian Academy of Sciences (SB RAS, Novosibirsk, Russia). All experiments in this study were approved by the Institutional Review Board and performed in accordance with Animal Care Regulations of ICG and with the international norms for studies with laboratory animals. At the age of 4 weeks, the pups were taken away from their mothers and housed in groups of five animals per cage (57×36×20 cm) and kept under standard laboratory conditions (22±2°C ambient temperature, 60% relative humidity, and natural light), provided with a standard rodent feed, PK-120-1, Ltd. (Laboratorsnab, Russia), and given water *ad libitum*.

Ophthalmoscopic examination was carried out using Beta direct ophthalmoscope (Germany) equipped with a slit lamp, after dilatation with 1% tropicamide. Ophthalmoscopic examination of rats of the parental strains (OXYS and WAG), second-generation (F2) hybrids and congenic animals was performed at ages 1.5-2 and 3-4 months. All animal procedures were in compliance with the Association for Research in Vision and Ophthalmology Statement for the Use of Animals in Ophthalmic and Vision Research as well as the European Communities Council Directive No. 86/609/EES. Assessment of stages of cataract and retinopathy was carried out according to the Age-Related Eye Disease Study (AREDS) grade protocol (http://eyephoto.ophth.wisc.edu). The following grades of cataract were used: 0 – transparent lens; 1 – very light cortical or nuclear opacity in the lens (corresponding to the decimal scale of the standard 1-4); 2 – opacity zones (corresponding to the decimal scale of the standard 5-8); 3 – intensive cortical or nuclear opacity of the lens (corresponding to the decimal scale of the standard 9-10). The degree of retinopathy was estimated as follows: 0 – arbitrary unit corresponding to healthy retina; 1 – appearance of drusen and other pathological changes in the retinal pigmented epithelium and partial atrophy of the choroid capillary layer; 2 – exudative detachment of RPE and of retinal neuroepithelium, with further choroid capillary layer atrophy.

### DNA extraction

DNA was extracted from tail tips using proteinase K and phenol extraction using standard methods. DNA was then reprecipitated and dissolved in deionized water, and DNA concentration was measured using Eppendorf Biophotometer (Germany).

### Microsatellite markers and polymerase chain reaction (PCR) on rat genomic DNA

Microsatellite markers were selected from the http://www-genome.wi.mit.edu and http://www.well.ox.ac.uk databases. The relative positions of markers on chromosomes, expressed in millions of nucleotides (megabases, Mb), were determined using the rat genome sequence at http://www.ensembl.org/. Twenty six microsatellite markers located on the rat chromosome 1 were tested. Of these only 13 showed interstrain polymorphism and intrastrain homogeneity during the testing of genomic DNA from OXYS and WAG rats. These markers were used in analyses of DNA from the population of F2 hybrids at 3-4 months of age (males and females). The ratio 1:2:1 as predicted by theory held for only 5 of 13 selected markers when we calculated the three marker classes ratio (homozygote by the first allelic variant, homozygote by the second allelic variant, and heterozygote), as estimated by χ^2^ criterion. These 5 markers were used in the following QTL-analysis: D1Rat196 (55.1 Mb), D1Rat224 (90.3 Mb), D1Rat30 (100.6 Mb), D1Rat219 (188.0 Mb), and D1Rat81 (250.4 Mb). The three markers that are located within the chromosomal regions of interest (D1Rat54, D1Rat117, and D1Rat76) were used to select the most appropriate animals in the backcross population for producing the next generation. The primer sequences for the markers used are given in Table 3.

**Table 3:** Primers used for analysis of chromosome 1

Marker	Annealing temperature, °C	Primer sequence, 5'→ 3'	Position on 1^st^ chromosome, Mb	Expected length of PCR fragment, bp
D1Rat150	64	AGAGGCAATGAAGTCCCTGA ATCCAGTGTCAACCTTTGGC	15.8	238
D1Rat234	62	GGGTACACTGGACTGGGAAA GCTGCCATTTAGTCTGGCTT	41.2	152
**D1Rat196**	62	TGCTTTTCAGATTCTCCTCC TACTGAGTCAATTTCTCTTGG	55.1	177
**D1Rat224**	58-64	AAAGCAATCTGTTTAAAAACAGTCA GCGTTTTCTCTGTCGCAATT	90.3	167
**D1Rat30**	64	AATTTCTGTCCCACATTTCCC TTCCAGGGACAAGCTACCTG	100.6	223
D1Rat183	64	CAGAAGCAAGCACACCAGTC TGTATTGGCTGGGAAGTTGG	131.2	226
D1Rat131	64	TCTGCGACTACCTTGGGTTT CCAGCAATTGAAATAACATTTTCA	145.7	176
D1Rat54	64	CTGACGGAAAAAAGGACAGG GTCTGCCTGCTGGGATTAAG	168.0	173
**D1Rat219**	60-58	GGAAGGGATCACATTGCATT GCAAAAGGACCTGTTGAAGC	188.0	248
D1Rat287	62-60	GTGCTATGGTGGGCAAGTTT GGGCGTGACCAGGTTACTTA	190.3	211
D1Rat168	64	AAGGAGCCACTAACTGTTCCC TCTCCAAAGCGGCTGAGTAT	204.8	210
D1Rat117	64	CCATGAGTTGCCATGGCTAT AATGCCACACAGAGAAGGGT	219.8	125
D1Rat76	64	GAAAGAGTGTGCGTGTGCAT CTTCTGTCTCCTCGCCAATC	230.6	144
**D1Rat81**	64	TGGTTCCAATGGATACCCATA TGAAGACTGAATCCCCCAAC	250.4	169
D1Rat86	64	AATACATGATGCTGTGGATTGG ACCCATTCCCACACCTGTAC	259.4	143
D1Wox19	58-70	TGTAATGGAATCTGATGCCC GGGCTCTATAGATAGGAGGTTTTAT	185.7	160
D1Arb31	68	AGAATTAAGTGGGAGGCTGGGC AGAGGATAAAGGAAGGGCGTGG	64.2	294
D1Mco17 (Atp1a3)	66	TGAGCTTCTGGTTGAAGGATCG CTCCACATATACCACCAAAGGC	80.3	165
D1Rat1	60	GCAATGCCATGGGTTTACTC AAAAGTTATCCCCTTCCCCC	10.6	129
D1Rat15	62-60	TGGAATGAAGGGGCTTACTG GTACAGGATGGCACTCGGTT	37.8	157
D1Rat27	60	TCTCTCCAGCTGCAGGATTT GGGCAAGCAAAGTACATGGT	90.3	177
D1Rat173	60-58	GATGGAGGCAGTTTTTCCAA GATCCCTTGACAAGCATGGT	145.3	156
D1Rat259	62	GTGGAACAGAGGGACTGCTT GCTTCCCTTCTCTGTGTTGAA	77.15	203
D1Rat28	60	ATGCACTCTATGATTGGCCC TGTCAGGACACATTCCTGCT	88.9	148
D1Mgh9	62-66	TGACCTCCACACGTGCTAAG AGAATGCTCAGGAAAAGTTAGGG	176.9	140
D1Rat195	58-60	CCCAGCATCAACCTCTTCC TTAACCTGCTTGGTTTTGGG	264.8	210

The five primers selected for QTL analysis are marked in bold. Legend: Mb – megabases; PCR – polymerase chain reaction; b.p. – base pairs.

PCR was performed in 1 × buffer (67 mM tris-HCl (pH 8.9), 16 mM (NH_4_)_2_SO_4_, 1.5 mM MgCl_2_, 0.01% Tween 20, and 10 mM β-mercaptoethanol) containing each of the four dNTP at 200 μM, each primer at 3 μM, with the addition of 50-100 ng of DNA, and 1 activity unit of Taq I DNA polymerase (Institute of Cytology and Genetics, SB RAS, Novosibirsk, Russia) in 25 μl reaction volume. Cycling conditions were as follows: initial denaturation at 95°C for 5 min, followed by 38 amplification cycles of denaturation at 95°C for 15 sec, primer annealing for 20 sec, and elongation at 72°C for 20 sec. The final elongation was 72°C for 5 min. We conducted a preliminary experiment to define optimal annealing temperature for each primer pair (shown in Table 3).

### Electrophoretic analysis of PCR fragments

PCR fragments were analyzed by electrophoresis in 6% polyacrylamide gels. Electrophoresis was performed in tris-borate buffer (0,5×TBE) at a field strength of 10 V/cm. Separated PCR fragments were visualized by EtBr staining.

### Statistical and linkage analysis

The data were analyzed using repeated measures ANOVA with the statistical package Statistica 6.0 (StatSoft, Tulsa, USA). Two-way ANOVA was used to assess gender differences in genotypes (sex × genotype). A Newman-Keuls *post hoc* test was applied to significant main effects and interactions in order to estimate the differences between particular sets of means. One-way ANOVA was used for individual group comparisons. Results were considered statistically significant if *p* value was less than 0.05.

Linkage of markers with hypothetical genes controlling the trait of interest was assessed by calculating the plausibility criterion (odds ratio) for and against linkage. The plausibility criterion for QTL is the LOD score (logarithm of odds). Linkage was considered significant when the LOD score was greater than the threshold value of 3.0. Linkage analysis was performed using the MAP MAKER/EXP 3.0 and MAPMAKER/QTL 1.1 programs (Whitehead Institute, Cambridge, MA) [15, 16].
